# Correlates of the Militant Extremist Mindset

**DOI:** 10.3389/fpsyg.2020.02250

**Published:** 2020-09-02

**Authors:** Adrian Furnham, George Horne, Simmy Grover

**Affiliations:** ^1^BI Norwegian Business School, Oslo, Norway; ^2^Department of Psychology, University of Bath, Bath, United Kingdom; ^3^Department of Experimental Psychology, University College London, London, United Kingdom

**Keywords:** militant extremism, Big Five, personality disorders, self-evaluations, militant, disorders affecting the musculoskeletal system

## Abstract

This study aimed to examine bright- and dark-side personality, personal beliefs (religion and politics) and self-evaluation correlates of beliefs in the Militant Extremist Mindset (MEM). In all, 506 young adults completed various self-report measures in addition to the three-dimensional MEM questionnaire. The measures included short measures of the Big Five traits, Self-Monitoring, Self-Evaluation and Personality Disorders, as well as demographic questions of how religious and politically liberal participants were. The Proviolence, Vile World, and Divine power mindsets showed varying correlates, with no consistent trend. Stepwise regressions showed that the demographic, personality and belief factors accounted for between 14% (Vile World) and 54% (Divine Power) of the variance, There were many differences between the results of three mindset factors, but personality disorder scores remained positive predictors of all three. The Vile World mindset was predicted by religiousness, liberalism, personality disorder scores and negative self-monitoring, but not personality traits. Religiousness had a contribution to all subscales and predicted the vast majority of the Divine Power mindset with smaller relationships with personality and personality disorders. Proviolence was predicted by the majority personality measures and sex.

## Introduction

For over 70 years personality and social psychologists have been developing attitude tests to measure political, social and religious attitudes. There are measures of authoritarianism, conspiracy theories, conservatism, dogmatism, ethnocentrism, fascism, Machiavellianism, paranoia, racism, social dominance etc. (Robinson et al., 1999). Many tests, and the theories from which they are derived, reflect the issues of the day and their particular time period. Hence, the interest in authoritarianism after the second war (Adorno et al., 1950), conservatism and racism in the 1960s (Wilson and Patterson, 1968; Eysenck and Wilson, 1978) and conspiracy theories today (Swami and Furnham, 2014). This study is about militant extremism which has attracted a number of recent studies (Loza, 2007; Trip et al., 2019; Gøtzsche-Astrup, 2020).

There appears to be a great deal of overlap in these theories and measures which suggest they would be highly correlated. An overview of two areas show to what extent they are related to the central theme of this study: *Militant Extremist Mindset.* The Authoritarian Personality (Adorno et al., 1950) focused on the individual as a cause of social evils. Authoritarians are nearly always ethnocentric in that they have a certain, simple and unshakable belief in the superiority of their own racial, cultural, and ethnic group with a powerful disdain for all those in other groups. They are characterized by three things: A strong desire to reject all ideas opposed to their own; A low degree of connectedness among various beliefs; Many more complex and positive ideas about things/issues they do believe in as opposed to those they don’t believe in.

Recent work in this area is exclusively on *Right-Wing Authoritarianism* (RWA) because it is recognized that left wing people like Stalinists and Trotskyists can equally be authoritarian. The idea is that RWA is made of up three attitudinal and behavioral clusters. The first is *Total Submission* to established authorities; the *Generalized Aggression* to all “enemies” of those authorities and the third *Blind Adherence* to established social norms and conventions. Thus, those with strong RWA beliefs tend to be absolutists, bullies, dogmatists, hypocrites and zealots (Altemeyer, 1998).

Another relevant, recent and related area of research is that of belief in *Conspiracy Theories* which are beliefs that attribute the ultimate cause of an event, or the concealment of an event from public knowledge, usually to a secret, unlawful, and malevolent plot by multiple actors working together (Sunstein and Vermeule, 2009; Swami et al., 2010). It has been suggested that there are four key characteristics that distinguish conspiracy theorists from real conspiratorial politics. First, conspiracy theorists consider the alleged conspirators to be Evil Incarnate. Second, they perceive the conspiratorial group as both monolithic and unerring in the pursuit of its goals: there is a single conspiratorial hub, which plans and coordinates its activities, and which possesses a high degree of internal solidarity, cohesiveness, and single-mindedness. Third, most conspiracy theories postulate the existence of a group of conspirators that is international and continuous in its temporal dimensions therefore to be capable of operating anywhere. Fourth, they believe that the conspiratorial group is “virtually omnipotent.” They form part of a “monological belief system” where once an individual has adopted anyone (major) conspiracist worldview, new conspiracy theories are assimilated more easily because they support that particular worldview.

### Militant Extremist Mindset

This study explores concepts of a relatively new concept: The Militant Extremist Mindset (MEMS), which has characteristics similar to those referenced above There appear to be three groups of researchers currently working in the area: Saucier in America, Stankov in Australia, and Knezevic and Mededovic in Serbia. Indeed, it was Knezevic in Serbia, together with Stankov and Saucier who first created the model, later joined by Mededovic (Saucier, 2000; Meedović and Petrović, 2016; Stankov and Lee, 2008, 2016a, b; Stankov, 2018; Stankov et al., 2018).

Most researchers in this area acknowledge the Saucier et al. (2009) paper as the seminal work in the area. According to Stankov et al. (2010a, b) the concept and questionnaire were constructed using three different methodologies: (a) Linguistic analyses based on a linguistic survey. (b) Conceptual analyses of terrorist texts. (c) Conceptual analysis of terrorist texts supplemented by literary and psychological sources on terrorists’ behavior, many of which being from Islamic terrorism.

The following 20 themes were extracted by Knezevic, Radovic and Milovanovic from these sources: Sacral Machiavellianism; Puritanism; Readiness for self-sacrifice; Manichaeism; Belief in life and reward after death; Anti-capitalism, anti-modernism, anti-democratism; Desire to be recognized by others; The feeling of anomie; Anti-rationalism and anti-hedonism; Intolerance of differing views; Feelings of repression and injustice; Revenge and the need to correct injustice; The feeling that one’s group is special; Propensity for taking action rather than thinking and deliberating; Hostility toward moderate people and moderate means; Cynicism about traditional ethics; Inability to decenter; Devaluation of the enemy; Chiliasm (millenarianism); Elimination of the differences between enemies.

A decade ago, Stankov et al. (2010a, b) developed a 24-item, three-dimensional test of MEMs. Factor1*: Proviolence*. This factor has loadings from 10 statements which indicates the acceptance of, justification, and even advocacy of the use of violence in certain circumstances like revenge or to gain redemption. Factor 2: *Vile World.* This factor has loadings from six statements, all of which indicate that there is something importantly wrong with the world we live in. Factor 3: *Divine Power.* This factor has eight statements, the most salient are those that make reference to a divine power, heaven and God. Another couple of themes have to do with the role of martyrdom and pleasures that will be bestowed on a person in the afterlife. Recent research has partly confirmed the factor structure of this measure (Stankov et al., 2019).

In this exploratory study we are interested in ideological (religious and political beliefs) correlates of the MEM. To a large extent, MEM beliefs reflect extreme religious beliefs and political orientation, hence we would expect them to be related. However, the size of the direction would be dependent on the three factors.

Based on these values and although this was an exploratory study we tested the following hypothesizes that MEM beliefs would be positively associated with religiousness but negatively associated with political liberalism. We expected that religious beliefs would be most closely related to the Divine Power factor and the political beliefs to the Proviolence factor.

More recently, Meedović and Knežević (2019) investigated whether the MEMS could be explained by Psychopathy, Sadism, and Disintegration as subclinical manifestations of amoral, antisocial, and psychotic-like traits. They showed that sadistic and psychopathic tendencies were related to Proviolence (advocating violence as a means for achieving a goal); psychopathic and disintegrative tendencies were associated to the Vile World (belief in a world as a corrupted and vile place), while Disintegration was the best predictor of Divine Power (relying on supernatural forces as a rationale for extremist acts). Vile World was found to be associated with stronger negative emotions as a response to violence.

In this study we looked at individual difference correlates on the MEMS. Usually when devising a new measure, researchers look for convergent and divergent validity by correlating the test with other well-established measures like the Big Five. In this study we used five measures to explore to what extent these factors could explain. We also used the Self-Monitoring scale which has been shown to be related to many social attitudes. It has been shown to be related to emotional intelligence, political and social skills as high self-monitors are able to adapt their behavior to social situations making them socially inconsistent. They tend not to be ideological in any sense adapting their views to the situation they find themselves in.

There is an extensive literature on personality and political beliefs (Furnham and Cheng, 2019; Furnham and Fenton-O’Creevy, 2019). The studies have consistently shown that all traits, particularly Agreeableness and Openness are associated with more left-wing and liberal views and participation in political events.

On the basis of research in the area we predict that all three MEM beliefs would be positively associated with Neuroticism but negatively associated with Openness Conscientiousness, Agreeableness and Self-Monitoring. We were particularly interested in how the personality dimensions were differentially associated with each Mindset but did not have any specific hypotheses.

In addition to these we used as short measure of the Personality Disorders. There has recently been a great deal of interest in the classification, measurement, and consequences of having sub-clinical and clinical personality disorders (Furnham, 2018, 2020; Harrison et al., 2018). Personality Disorders are defined as inflexible, maladaptive, and persisting beliefs and behaviors which cause significant functional impairment or subjective distress. It is the inflexible, repetitive, poor stress-coping responses that are marks of disorder. Most find it very difficult to establish and maintain long-term happy health relationships. Hence, we hypothesized that scores on the personality disorder measure would positively correlate with MEM scores.

Finally, we included a simple short measure of self-evaluations. There is an extensive literature from various areas of psychology from the extensive work on Core Self-Evaluations (Judge and Bono, 2001) to Self-Assessed intelligence and attractiveness (Swami et al., 2007; Kornilova et al., 2009; Furnham and Grover, 2020) that suggests that positive self-evaluations are psychologically healthy with important behavioral consequences. In short, people who feel good about themselves tend to have a more benevolent and less malevolent view of others. In this study, we predicted that positive self-evaluations would be significantly negatively correlated with the MEM facets.

## Materials and Methods

### Participants

In all there were 506 adults of which 291 were male and 215 females. They were on average 20.34 years old (SD = 3.57), and many were in higher education. Just over a fifth (21.5%) had a high school certificate as their highest qualification, nearly a half (46.4%) an undergraduate degree and just over a quarter (28.3%) had a post-graduate degree. In all 58% were single, 19.6% married and 19.8% co-habiting. Overall, 78% were child-free; 11% had 1 child and 7.1% 2 children. Just under a quarter (24%) were monolingual; 41.4% bi-lingual and 18.2% trilingual. On a scale of 0 (Not at all) to 9 (Very) they rated their religiousness as 2.22 (SD = 2.74) and 61.64% noted they did not Believe in Life-after-Death, while 37.8% did. They also rated themselves on a 9 point (1 = Very Conservative; 9 = Very Liberal) political beliefs scale where the mean was 6.10 (SD = 1.71).

#### Questionnaires

1. *Militant Extremist Mindset Questionnaire* (MEMS; Stankov et al., 2010a, b). The Proviolence scale has 10 items (Alpha 0.80), the Vile World scale has six items (Alpha 0.85) and the Divine Power scale has eight items (Alpha 0.78). All of the scales included in the research use a standard 7-point Likert response scale.
2. *Ten Item Personality Inventory* (Gosling et al., 2003). The TIPI includes 10 items that assess the Big Five personality factors. Participants were asked the extent to which a pair of traits applied to them and rated on a 7-point scale (from 1 = disagree strongly to 7 = agree strongly). Hundreds of studies have used this short measure which correlates highly with longer measures of the same concepts (Furnham, 2008).
3. *Self-Monitoring Scale*: (Snyder and Gangestad, 1986). This is an 18-item scale and in this study was measured on an 7 point agree disagree scale (Alpha 0.79). Various factor analyses have been done with slighly different results. Though correlated with personality measures the test is a good predictor of the size and complexity of a individual’s personal network (Grover, 2018).
4. *Personality Disorders Questionnaire* (Lange et al., 2012). This is a self-administered screening questionnaire that includes 12 items from the Personality Self Portrait (Oldham and Morris, 1990) (Alpha 0.81). It showed theoretically expected associations with membership in different subsamples and is a new instrument for identifying different classes of personality disorder severity already at the screening stage of the diagnostic process.
5. *Self-Evaluations* (Furnham, 2018). Participants rated themselves on a number of factors on a 100-point scale for four characteristics: Attractiveness, Physical Health, Intelligence and Emotional Intelligence. These measures were combined to form a single self-esteem measure which had (Alpha 0.79).

#### Procedure

Ethics committee (CEHP/514/2017) permission was sought and received. Participants were recruited online using the Prolific platform. They were told their anonymous results would be used for analysis and paid £1.00 for their participation. The test took on average 8 min to complete. A small number of participants (around 3%) had incomplete cases were excluded from further analysis.

## Results

We set out first to examine the correlation between all variables and secondly, through regression analysis to examine the relative contribution of our independent variables to beliefs in the three MEM scales. The correlations between the three MEM scores was low 0.06 < r < 20 indicating they were independent of each other.

### Correlation Analysis

All variables were found to be non-normal using Shapiro-Wilk tests. Therefore, Spearman’s rho correlation coefficients were used to measure the association between variables to produce more accurate coefficients.

Table 1 shows the Proviolence mindset had negative associations with Openness, Conscientiousness, and Agreeableness. Proviolence was also the only mindset significantly correlated with gender, showing it to be greater in men and the only mindset associated with self-monitoring. Proviolence was positively associated with personality disorder scores, but not as strongly as Vile World subscale. The Proviolence subscale also had a small but significant relationship with religiousness and a negative association with liberalism.

**TABLE 1:** Spearman’s Rho correlation table with MEM subscales.

	Mean	SD	α	1	2	3	4	5	6	7	8	9	10	11	12	13	14
1. Sex																	
2. Age	32.75	87.5		0.17***													
3. Religiousness	2.21	2.74		0.01	0.06												
4. Liberalism	6.11	1.78		0.11*	–0.09	−0.32***											
5. Openness	10.08	2.26	0.37	0.01	–0.02	–0.00	0.15***										
6. Conscientious	9.92	2.23	0.50	0.14**	0.12**	–0.04	0.02	0.10*									
7. Extraversion	7.07	2.97	0.68	0.04	0.04	0.03	0.15***	–0.05	0.26***								
8. Agreeable	9.37	2.23	0.30	0.19***	0.06	0.05	0.05	0.07	0.22***	0.01							
9. Neuroticism	7.48	2.82	0.69	0.18***	–0.09	–0.09	0.05	−0.14***	−0.25***	−0.22***	−0.15***						
10. SMS Total	86.39	14.4	0.72	−0.11**	−0.17***	0.02	0.09	0.16***	−0.04*	0.369***	–0.02	0.01					
11. PDS Total	47.47	12.2	0.81	0.05	−0.15***	–0.09	0.01	−0.12**	−0.27***	−0.22***	–0.20	0.39***	0.11*				
12. MEM Prov	2.15	0.930	0.82	−0.18***	−**0.07**	0.11*	−0.20***	−0.15***	−0.23***	−**0.00**	−0.23***	**0.06**	0.17***	0.20***			
13. MEM Vile	4.36	1.29	0.845	**0.05**	−**0.08**	**0.01**	**0.06**	−**0.02**	−**0.06**	−0.10*	−0.10*	0.16***	−**0.06**	0.33***	0.02		
14. MEM DP	2.91	1.20	0.82	**0.03**	−**0.01**	0.70***	−0.33***	−**0.06**	−**0.06**	0.15***	0.10*	−0.09*	**0.07**	**0.02**	0.23***	0.00	
15. Self-Evalua	67.85	13.9	0.71	–0.08	0.02	0.10*	0.12***	0.27***	0.23***	0.30***	0.09*	−0.30***	0.16***	−0.32***	−0.09*	−0.12**	**0**.**040**

***p < 0.001, **p < 0.01, *p < 0.05. Sex coded as Female = 1, Male = 2.

Alternatively, Vile World mindset had no significant associations with Openness, Conscientiousness nor self-monitoring, but instead a negative association with Extraversion and a positive association with Neuroticism There was a negative association with Agreeableness and self-evaluations. Vile World was the only mindset of the three to be unassociated with religiousness.

The Divine Power subcale was the most unlike the others subscales, having positive association with Extraversion and Agreeableness. Notably, this subscale had no association with personality disorders, self-monitoring or self-evaluation scores. Instead, it had a very strong association with religiousness and the strongest (negative) association with liberalism.

#### Regression Analyses

Hierarchical regressions were used to analyze incremental validity and show the relationship between the predictor variables (Pedhazur, 1997). For each regression, the first step added sex, age, self-reported religiousness and liberalism variables as predictors; the second the Big Five personality traits; and the third step added self- monitoring, personality disorder and self-evaluation scores (see Table 2).

**TABLE 2:** Multiple regressions with MEM scale and subscales.

	Step 1		Step 2		Step 3	
	β	*t*	β	*t*	β	*t*
***Proviolence***						
Sex	–0.152	−3.30**	–0.114	−2.45*	–0.108	−2.36*
Age	0.000	0.01	0.015	0.33	0.049	1.12
Religiousness	0.077	1.61	0.094	2.00*	0.105	2.30**
Liberalism	–0.151	−3.13**	–0.108	−2.28*	–0.110	−2.35*
Openness			–0.172	−3.71***	–0.176	−3.86***
Conscientiousness			–0.140	−3.01**	–0.090	–1.92
Extraversion			0.044	0.94	0.018	0.34
Agreeableness			–0.164	−3.56***	–0.141	−3.17**
Neuroticism			–0.004	–0.07	–0.076	–1.56
SMS Total					0.157	3.24**
PDS Total					0.178	3.55***
Self-Evaluations					–0.038	–0.77
F	7.91***		8.88***		9.61***	
Adj R^2^	0.057		0.134		0.183	
***Vile World***						
Sex	0.092	1.96	0.080	1.65	0.037	0.79
Age	–0.107	−2.27*	–0.087	–1.85	–0.086	–1.90
Religiousness	0.091	1.86	0.119	2.44*	0.133	2.83**
Liberalism	0.089	1.81	0.088	1.78	0.119	2.47*
Openness			0.030	0.62	0.041	0.87
Conscientiousness			–0.006	–0.12	0.041	0.86
Extraversion			–0.058	–1.19	0.060	1.15
Agreeableness			–0.095	−2.01*	–0.051	–1.12
Neuroticism			0.162	3.25**	0.075	1.49
SMS Total					–0.148	−2.96**
PDS Total					0.331	6.44***
Self-Evaluations					–0.023	–0.45
F	3.22*		3.88***		7.08***	
Adj R^2^	0.019		0.053		0.137	
***Divine Power***						
Sex	0.045	1.37	0.039	1.16	0.038	1.10
Age	–0.057	–1.72	–0.062	–1.88	–0.049	–1.48
Religiousness	0.694	20.18***	0.676	19.75***	0.682	19.99***
Liberalism	–0.096	−2.79**	–0.087	−2.51*	–0.084	−2.41*
Openness			–0.088	−2.59**	–0.088	−2.58*
Conscientiousness			–0.039	–1.16	–0.016	–0.45
Extraversion			0.046	1.37	0.045	1.18
Agreeableness			0.104	3.14**	0.116	3.48***
Neuroticism			–0.051	–1.45	–0.085	−2.31*
SMS Total					0.050	1.39
PDS Total					0.087	2.33*
Self-Evaluations					–0.023	–0.63
F	126.31***		60.19***		46.67***	
Adj R^2^	0.521		0.537		0.544	

***p < 0.01, **p < 0.01, *p < 0.05.

Demographic and personality variables explained 18% of the variance in the Proviolence subscale. Sex was a significant predictor in the first step, with males having higher scores along with liberalism. In the second step, Openness, Conscientiousness and Agreeableness were all negative predictors, and after adding the Big Five personality factors, religiousness emerged as a significant predictor In the third step, Conscientiousness lost its significance and self-monitoring and personality disorder scores were positive and significant.

The Vile World subscale had the least variance explained accounted for by the predictors, at 14% Age was the only significant negative predictor in the first step. However, after adding the five-factors of personality in the second step, age lost its significance, and religiousness became significant. Agreeableness negatively and Neuroticism positively were also significant predictors. In the final step the two personality factors above lost their significance, while liberalism gained significance. Self-monitoring and personality disorder scores were also significant negative predictors.

The Divine Power subscale was the most predicted of all the subscales with over 54% of its variance explained in the final step. Religiousness positively and liberalism negatively were both significant predictors in the first step. Openness positively and Agreeableness negatively were significant predictors in the second step Personality disorders was also a positive significant predictor in the third step and Neuroticism emerged as a significant negative predictor.

## Discussion

Our results show that Divine Power is the most predictable mindset, at least from our demographic and basic personality variables. Divine Power has a negative association with Neuroticism and Openness but a positive relationship with Agreeableness even after controlling for religiousness. One interpretation of these results is that more trusting and naïve people tend to fundamentalist religious views though this idea and finding warrant more exploration. Open individuals would be much less likely to be attracted to the rigidity and inflexibility of any fundamentalist creed.

Confirming the importance of religiousness to the MEMS questionnaire, two of the mindsets were positively predicted by religiousness: Vile world was the exception. This could be the result of the test’s creation and its focus on the religion (Islamic) as was current upon its creation (Stankov et al., 2010a, b). Note that in this study we asked how religious people were, not to which religion they adhered or indeed the precise nature of their beliefs (i.e., fundamentalist).

However, while Proviolence and Divine Power beliefs were negatively predicted by liberalism, Vile World was instead positively predicted by liberalism in the regressions. This may reflect the mindset of RWA outlined by Altemeyer (1998). Belief in a Divine Power maps onto *total submission* to established authority (the divine) and Blind Adherence could relate to the culture surrounding the religion and their norms. Proviolence relates to the *generalized aggression* toward enemies of those authorities. Vile World’s positive relationship with liberalism reflects is measurement, including items such as “*Modern governments have overstepped moral bounds and no longer have a right to rule*” and “*Evil has been re-incarnated in the cult of markets and the rule of multinational companies*” reflecting an anti-authoritarian and anti-capitalist beliefs, respectively.

Predictive relationships between Big Five personality traits varied across extremism mindsets. Neuroticism positively predicted Vile World mindsets in the second step of the model, but this prediction was lost when personality disorders and self-monitoring were added in step three, this suggests that these two variables may mediate the relationship between Neuroticism and Vile World mindsets. Conversely, Neuroticism emerged as a significant negative predictor for Divine Power mindsets in step three. These results only partially support our hypotheses, as Openness significantly predicts Proviolence and Divine Power, but not Vile World mindsets. Conscientiousness only negatively predicts Proviolence mindsets, but even then, is non-longer significant once more significant predictors, such as self-monitoring and personality disorders are added in the final step Agreeableness is more varied, negatively predicting Proviolence, and Vile World (in step two), but positively predicts Divine Power mindsets. The latter likely due to the community and prosocial teachings of institutionalized religion.

All mindsets were positively predicted by personality disorder scores, confirming our hypotheses. Of the three, personality disorders predicted Vile World mindsets most Self-monitoring also negative predicted the Vile World mindset. Vile World’s relationship with self-monitoring is unique amongst the three mindsets, as Proviolence has the inverse, being positively predicted by self-monitoring scores, and there being no relationship with Divine Power.

Somewhat surprisingly, self-evaluations significantly predicted none of the three mindsets when it was added in the last step. While it had significant negative correlations with Proviolence and Vile World, these disappeared when the other variables were controlled for. This suggests that self-evaluations may only have indirect effects on militant mindsets, through other personality measures.

A major limitation of this study was the measurement of Big Five personality through the TIPI (Gosling et al., 2003), a 10-item measure. Longer measures, such as the BFI-2 (Soto and John, 2017), might explain the MEM better, dividing the Big Five into three facets each. Some factors may only have one facet related to the mindset, or even two facets with opposing effects which may be leading to non-significant results with measurement. For example, although Extraversion had no significant prediction on Divine Power, the mindset may have been positively associated with Sociability one facet of Extraversion, due to religiousness people being more likely to be part of a religious community, while negatively associated with Assertiveness, another facet.

Further research on Western populations could use this measure (specifically the Proviolence and Vile World subscales) alongside other personality measures (dark and light) to investigate environmental and political activism, particularly racism.

## Data Availability Statement

The raw data supporting the conclusions of this article will be made available by the authors, without undue reservation.

## Ethics Statement

The studies involving human participants were reviewed and approved by CEHP/514/2017. The patients/participants provided their written informed consent to participate in this study.

## Author Contributions

AF collected the data and wrote the manuscript. GH and SG analyzed the data. All authors contributed to the article and approved the submitted version.

## Conflict of Interest

The authors declare that the research was conducted in the absence of any commercial or financial relationships that could be construed as a potential conflict of interest.
